# An Updated Meta-Analysis of the Efficacy and Safety of Prostatic Artery Embolization vs. Transurethral Resection of the Prostate in the Treatment of Benign Prostatic Hyperplasia

**DOI:** 10.3389/fsurg.2021.779571

**Published:** 2021-12-15

**Authors:** Zhunan Xu, Zhongbao Zhou, Yingmei Mu, Tong Cai, Zhenli Gao, Lingling Liu

**Affiliations:** ^1^Second Clinical Medical College, Binzhou Medical University, Yantai, China; ^2^Department of Urology, The Affiliated Yantai Yuhuangding Hospital of Qingdao University, Yantai, China; ^3^Department of Urology, Beijing TianTan Hospital, Capital Medical University, Beijing, China; ^4^Department of Allergy, The Affiliated Yantai Yuhuangding Hospital of Qingdao University, Yantai, China

**Keywords:** Benign prostatic hyperplasia, efficacy, safety, meta-analysis, systematic review

## Abstract

**Background:** Prostatic artery embolization (PAE) in the treatment of benign prostatic hyperplasia (BPH) has been introduced into clinical practice, but conclusive evidence of efficacy and safety has been lacking.

**Objective:** To compare the efficacy and safety of prostatic artery embolization (PAE) vs. transurethral resection of prostate (TURP), we performed a meta-analysis of clinical trials.

**Methods:** We searched randomized controlled trials (RCTs) from Pubmed, Embase, Wanfang, and CNKI from January 2000 to December 2020 and used RevMan 5.0 to analyze the data after five RCTs were included.

**Results:** The reducing of prostate volume (PV) [Median mean (MD) 14.87; 95% confidence interval (CI) 7.52–22.22; *P* < 0.0001] and the increasing of maximum flow rate in free uroflowmetry (Qmax) (MD 3.73; 95% CI 0.19–7.27; *P* = 0.004) were more obvious in TURP than in PAE; however, the rate of lower sexual dysfunction [odds ratio (OR) 0.12; 95% CI 0.05–0.30; *P* < 0.00001] was lower in PAE compared with TURP. Meanwhile, no conspicuous difference in International Prostate Symptoms Score (IPSS) score (MD 1.42; 95% CI −0.92 to 3.75; *P* = 0.23), quality of life (Qol) score (MD 0.21; 95% CI −0.31 to 0.73; *P* = 0.43), post void residual (PVR) (MD 21.16; 95% CI −5.58 to 47.89; *P* = 0.12), prostate-specific antigen (PSA) (MD 0.56; 95% CI −0.15 to 1.27; *P* = 0.12), and complications (OR 0.90; 95% CI 0.20–4.05; *P* = 0.89) between PAE and TURP group was shown.

**Conclusion:** PAE may replace TURP as an alternative treatment for Benign prostatic hyperplasia (BPH) patients who do not want to have surgery or with operational contraindications.

## Introduction

BPH is one of the most common illnesses amongst men and is connected with lower urinary tract symptoms (LUTS). The morbidity rate of BPH is more than 50% of men over 60 years old and it is positively correlated with age (1, 2). The main manifestations of BPH are pollakiuria, urgency, and progressive dysuria, which are also called LUTS, and about 60% of LUTS are caused by BPH in men aged 50- to 60-years-old (3).

Therapeutic protocols for BPH often include clinical observation, medication, minimally invasive procedures, or surgical treatment. The preferred medications are alpha-blockers and 5-alphareductase inhibitors (5-ARIs) (4, 5). Minimally invasive approaches include transurethral microwave thermotherapy, PAE, and transurethral laser vaporization therapies (6, 7). Surgical therapies include TURP, endoscopic enucleation, and open surgery. TURP is the current standard operative protocol for treatment of BPH with prostate volumes of 30–80 ml according to the European or American urology (EAU) guidelines; endoscopic enucleation and open prostatectomy (OP) are propitious for patients with large volumes (over 80–100 mL) (3, 4). However, current international guidelines offer endoscopic enucleation as a size-independent option and possibly a new standard. A high risk for surgical complications is associated with men more than 60 years old; they include urinary infection, strictures, postoperative pain, incontinence or urinary retention, sexual dysfunction, and blood loss (8). In order to reduce surgery-related complications, several minimally invasive treatments mentioned above have been proposed.

PAE is a minimally invasive interventional radiological procedure which could lead to ischemia or atrophy of the prostate through injecting microspheres or small particles into the prostatic arteries bilaterally or unilaterally. Initially, PAE was considered as a procedure to control hemorrhage after prostatectomy or prostate biopsy, then De Meritt et al. (9) accidentally discovered that the enlarged prostate volume could shrink and clinical symptoms could further relieve after PAE. Although PAE is offered as a standard therapeutic option for patients with BPH-LUTS in National Institute for Health and Care Excellence (NICE) and EAU guidelines (10, 11), TURP is still the gold standard. To date, only six RCTs have been published comparing the efficacy and safety of PAE with TURP (7, 12–16). We performed a meta-analysis to compare the effectiveness and safety of PAE vs. TURP.

## Methods

### Search Strategy

Systematic retrieval for relevant literature with the language restricted to Chinese or English up to September 2021 was performed in Pubmed, Embase, Wanfang, and CNKI. Search words consisted of “benign prostate hyperplasia or BPH,” “prostatic arterial embolization or PAE,” and “transurethral resection of the prostate or TURP”. Two reviewers browsed all articles independently, and only RCTs were selected for inclusion in the study. Furthermore, we also revised correlative studies.

### Criteria for Inclusion and Exclusion

We only included RCTs that analyzed the effectiveness and safety of PAE vs. TURP for treating BPH. The RCTs should involve effective data (such as IPSS, QOL, Qmax, PV, PVR, PSA, complications, and sexual dysfunction).

We excluded (1) reviews, comments, recommendations, letters, ongoing trials, protocols, meeting abstracts, consensus or statement and articles lacking applicable data; (2) studies with incomplete data, such as missing the standard deviation or number; (3) non-randomized comparative studies; and (4) non-comparative studies (case reports or case series).

### Data Extraction

Two researchers reviewed studies included and extracted data independently using a self-defined data sheet. The data we extracted included basic information (Author, Year, Country), methods, inclusion criteria, participants (patients' number), interventions (operational styles), and outcomes (evaluation points). We compared the postoperative outcomes after 12 months. If the follow-up time was <12 months, the statistical data nearest to 12 months was put into use for the analysis. The data researched in this meta-analysis were contrasts of post interventional mean changes in: (1) IPSS, (2) QoL, (3) Qmax, (4) PV, (5) PVR, (6) PSA, (7) complications, and (8) sexual dysfunction.

### Quality Assessment

We employed Revised Jadad composite scale (17–19) ranking from 0 to 7 points to assess the quality of RCTs from the following aspects: randomization, double blinding, concealment of allocation, and withdrawals and dropouts. “High-quality studies” were studies scoring ≥ 4 points (19). Everyone agreed with this result after all authors took part in the assessment of included studies.

### Data Analysis

Review Manager version 5.3 was employed to analyze the data. We used MD with the corresponding 95% CI to explain continuous data and OR for dichotomous results. If *p* < 0.05, the result of statistics was remarkable. The inconsistency was analyzed using *I*^2^ statistic that mirrored the proportion of heterogeneity in data analysis. A fixed effect model would be used for results where the *I*^2^ value is <50% and has insignificant heterogeneity. Otherwise, random effect model was applicated.

## Results

### Study Inclusion

**Figure 1:**
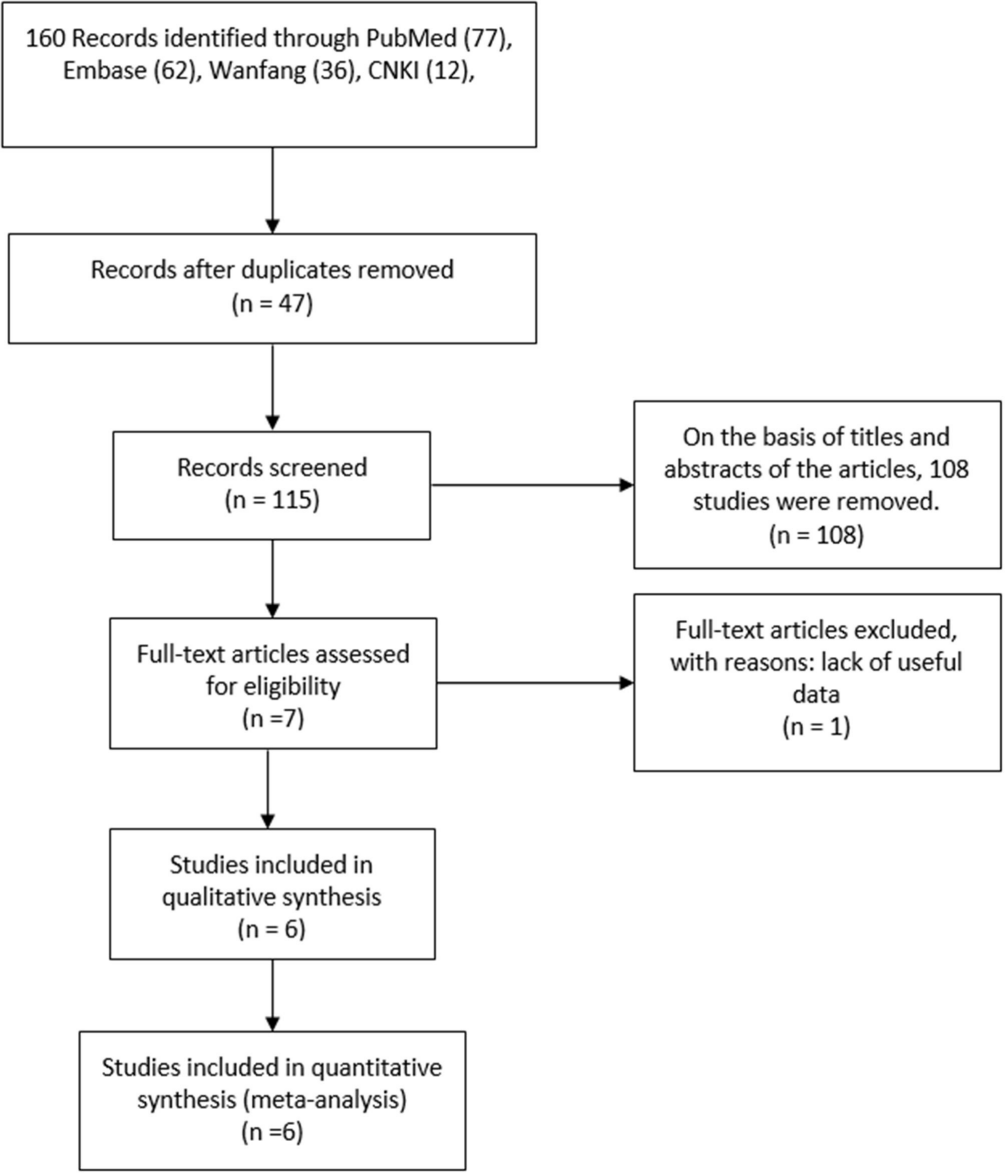
Flowchart of study inclusion.

Altogether 160 trials were selected through the searching procedure. From these trials, five RCTs (7, 12–16) that included 402 BPH participants (PAE: 194 participants and TURP: 208 participants) were chosen in our meta-analysis (Figure 1). The traits of six selected RCTs (7, 12–16) are revealed in Table 1. A brief overview of patients in the included trials is revealed in Table 2.

**Table 1:** General characteristics of the studies included.

**References, Country**	**Methods**	**Inclusion criteria**	**Participants**	**Intervention**	**Outcomes**
Abt et al. (7), Switzerland	*RCT*	Refractory BPH-LUTS; Age ≥ 40 year; IPSS ≥ 8; QOL ≥ 3; prostate size 25–80 ml; candidate for TURP; refractory to medical therapy or refuse to consider (further) medical treatment; Qmax < 12 ml/s and/or urinary retention; written informed consent	PAE:48 TURP:51	PAE: Bilateral or unilateral embolization; 250–400 um microspheres. TURP: Monopolar TURP	Changes in IPSS (time frame: 12 week) Changes in: Qmax; PVR; questionnaires IPSS, CPSI, and IIEF-5 (assessed at 1, 6, 12 week, 6, 12, 24, 60 month); changes in prostate volume (assessed at 12 week and 24 month); Complications and sexual dysfunction Only 3 months data available so far
Carnevale et al. (12), Brazil	*RCT*	Severe BPH-LUTS; Age > 45 year; IPSS > 19; refractory to medical therapy for at least 6 months; prostate size 30–90 ml; bladder obstruction; written informed consent	PAE:15 TURP:15	PAE: Bilateral embolization; 300–500 μm microspheres. TURP: Monopolar TURP	Changes at 12 month reported for: Qmax, PVR, IPSS; IIEF-5; PSA, prostate volume; complications and sexual dysfunction
Gao et al. (13), China	*RCT*	With moderate to severe LUTS due to BPH. IPSS >7; Failed medical therapy with 2-wk washout period; prostate volume 20–100 ml; Qmax <15 ml/s; Written informed consent	PAE:57 TURP:57	PAE: Bilateral or unilateral embolization; 355–500 μm polyvinyl alcohol microspheres. TURP: Bipolar TURP	IPSS; Qmax; PVR; prostate volume; PSA; complications (assessed at 1, 3, 6, 12, and 24 month); perioperative data including procedure time and radiation parameters
Zhu et al. (14), China	*RCT*	BPH-LUTS; patient without contraindication; without previous history of surgery; without taking 5-alpha reductase inhibitors 4 week before surgery; Written informed consent	PAE:20 TURP:20	PAE: Bilateral embolization; 100–300 or 310–500 μm Microspheres TURP: TURP	IPSS; QOL; *Q*max; PVR; prostate volume; PSA; complications and sexual dysfunction (assessed at 3, 6, 12 month)
Insausti et al. (15), Spain	*RCT*	age >60 years; BPH-related LUTS refractory to medical treatment for at least 6 months or the patient could not tolerate medical treatment; TURP was indicated; the International Prostate Symptom Score (IPSS) was ≥8; quality of life (QoL) related to LUTS was ≥3; and the peak flow rate (Qmax) was ≥10 mL/s or urinary retention.	PAE:23 TURP:22	PAE: Bilateral embolization; 300–500 um microspheres. TURP: Monopolar TURP	Changes in IPSS; Qmax; PVR; PV; QOL; complications (assessed at baseline and at 3, 6, and 12 months); Changes in PSA (assessed at baseline and at 3 and 12 months)
Abt et al. (16), Switzerland	RCT	Refractory BPH-LUTS; Age ≥ 40 year; IPSS ≥ 8; QOL ≥ 3; prostate size 25–80 ml; candidate for TURP; refractory to medical therapy or refuse to consider (further) medical treatment; Qmax <12 ml/s and/or urinary retention; written informed consent	PAE:34 TURP:47	PAE: Bilateral or unilateral embolization; 250–400 um microspheres. TURP: Monopolar TURP	Changes at 3, 6, 12, 24 month reported for: IPSS, Qmax; PVR; questionnaires IPSS, CPSI, IIEF-5, prostate volume, Complications and sexual dysfunction

*BPH, Benign prostatic hyperplasia; IIEF-5, International Index of Erectile Function; IPSS, international prostate symptom score; LUTS, lower urinary tract symptoms; MRI, magnetic resonance imaging; PAE, prostatic artery embolization; PSA, prostate-specific antigen; PVR, Post-void residual urine; Qmax, urinary peak flow; QOL, quality of life; RCT, randomized controlled trial; TURP, transurethral resection of the prostate*.

**Table 2:** Brief overview of patients in the included trials.

**Trial, year**	**Age**		**Baseline prostate volume**	
	**PAE**	**TURP**	**PAE**	**TURP**
Abt 2018 (7, 16)	65.7 (9.3)	66.1 (9.8)	52.8 (32)	56.5 (31.1)
Carnevale 2016 (12)	63.5 (8.7)	66.4 (5.6)	63 (17.8)	56.6 (21.5)
Gao 2013 (13)	67.7 (8.7)	66.4 (7.8)	64.7 (19.7)	63.5 (18.6)
Zhu 2018 (14)	61.1 (4.4)	62.4 (4.9)	81.21 (6.34)	82.09 (6.47)
Insausti 2000 (15)	72.4 (6.2)	71.8 (5.5)	60 (21.6)	62.8 (23.8)

### Study Quality

According to the revised Jadad scale, there are three high-quality trials (score ≥ 4 points) (7, 14–16) and two poor-quality trials (score <4 points) (12, 13) (Table 3).

**Table 3:** Assessment of randomized study quality.

**Trial, year**	**Random sequence** **production**	**Allocation** **concealment**	**Blind method**	**Withdrawals**	**Score**
Abt 2018 (7)	Adequate	Unclear	Inadequate	Description	4
Carnevale 2016 (12)	Unclear	Unclear	Inadequate	Description	3
Gao 2013 (13)	Adequate	Inadequate	Inadequate	Description	3
Zhu 2018 (14)	Adequate	Unclear	Inadequate	Description	4
Insausti 2000 (15)	Adequate	Unclear	Inadequate	Description	4
Abt 2021 (16)	Adequate	Unclear	Inadequate	Description	4

### Result of Meta-Analysis

#### Efficacy

##### IPSS

**Figure 2:**
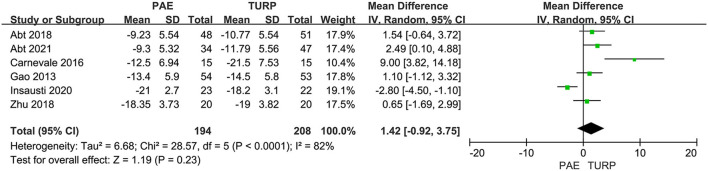
Forest plot for IPSS. IPSS, International Prostate Symptoms Score.

We used six RCTs involving the aggregate of 402 patients (PAE:194 participants and TURP:208 participants) to assess the variation of IPSS. There was obvious heterogeneity among these trials according to the analysis result (*I*^2^ = 82%; *P* <0.0001). The forest plot demonstrated that the difference of postoperative IPSS between TURP and PAE was not statistically significant (MD 1.42; 95% CI −0.92 to 3.75; *P* = 0.23; Figure 2).

##### QoL

**Figure 3:**
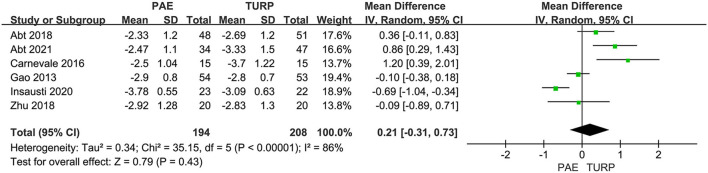
Forest plot for QoL. QOL, quality of life.

Six RCTs including 402 patients in total were employed to analyze the difference of QoL. According to the analysis data, heterogeneity among five trials was substantial (*I*^2^ = 86%; *P* <0.0001). The forest plot demonstrated that the difference of postoperative QoL between TURP and PAE was not statistically significant (MD 0.21; 95% CI −0.31 to 0.73; *P* = 0.43; Figure 3).

##### Qmax

**Figure 4:**
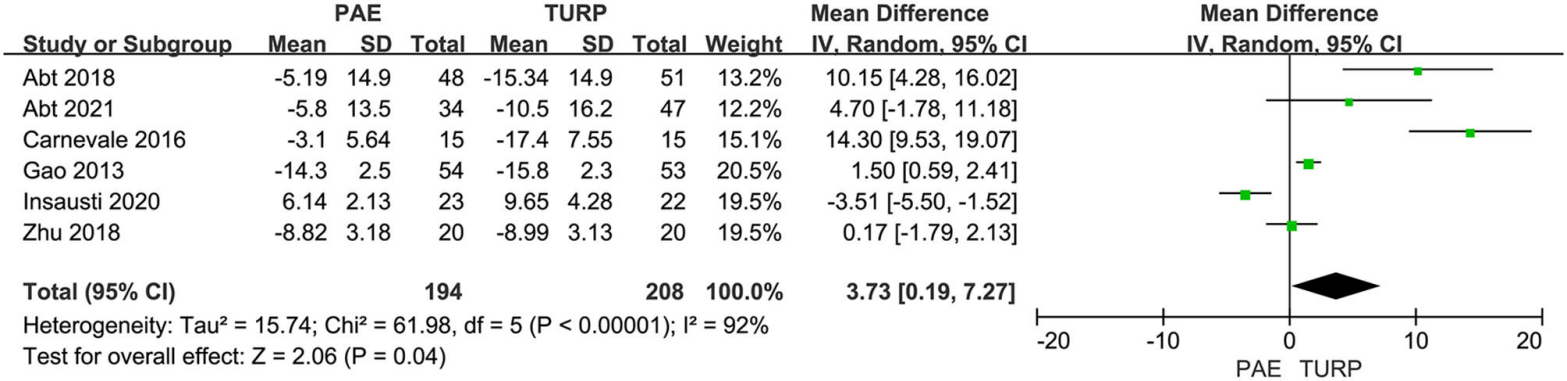
Forest plot for Qmax. Qmax, maximum flow rate.

Six RCTs comprising 402 patients were applied to assess the change of Qmax. There was obvious heterogeneity among these trials according to the analysis result (*I*^2^ = 92%; *P* <0.00001). The model showed that the increase of postoperative Qmax in TURP group was more significant than that in PAE group (MD 3.73; 95% CI 0.19–7.27; *P* = 0.004; Figure 4).

##### PV

**Figure 5:**
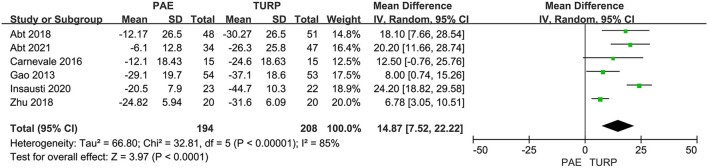
Forest plot for PV. PV, prostate volume.

We used six RCTs including 402 patients altogether to analyze the change of PV. Substantial heterogeneity was shown between these trials according to the analysis result (*I*^2^ = 85%; *P* <0.00001). The model demonstrated that the reductive level of PV in TURP group was more obvious than that in PAE group (MD 14.87; 95% CI 7.52–22.22; *P* <0.0001; Figure 5).

##### PVR

**Figure 6:**
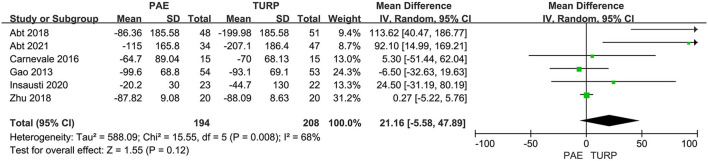
Forest plot for PVR. PVR, post void residual.

Six RCTs involving 402 patients in total were applied to assess the difference of PVR. There was obvious heterogeneity among these trials according to the analysis result (*I*^2^ = 68%; *P* = 0.008). The model revealed no significant difference for PVR between the PAE group and the TURP group (MD 21.16; 95% CI −5.58 to 47.89; *P* = 0.12; Figure 6).

##### PSA

**Figure 7:**
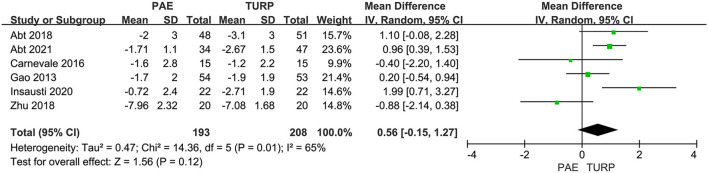
Forest plot for PSA. PSA, prostate-specific antigen.

Six RCTs made up of 402 patients were employed to evaluate the difference of PSA. A substantial heterogeneity was shown between these trials according to the analysis result (*I*^2^ = 65%; *P* = 0.01). The model revealed no statistical difference for the change of PSA between the PAE group and the TURP group (MD 0.56; 95% CI −0.15 to 1.27; *P* = 0.12; Figure 7).

#### Safety

##### Complications

**Figure 8:**
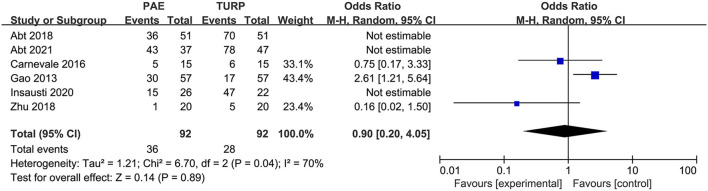
Forest plot for complications.

We used six RCTs with 402 patients altogether to analyze the rate of complications. There was obvious heterogeneity among these trials according to the analysis result (*I*^2^ = 70%; *P* = 0.04). No statistical difference was demonstrated for the rate of complications between the PAE group and the TURP group according to the model (OR 0.90; 95% CI 0.20–4.05; *P* = 0.89; Figure 8).

##### Sexual Dysfunction

**Figure 9:**
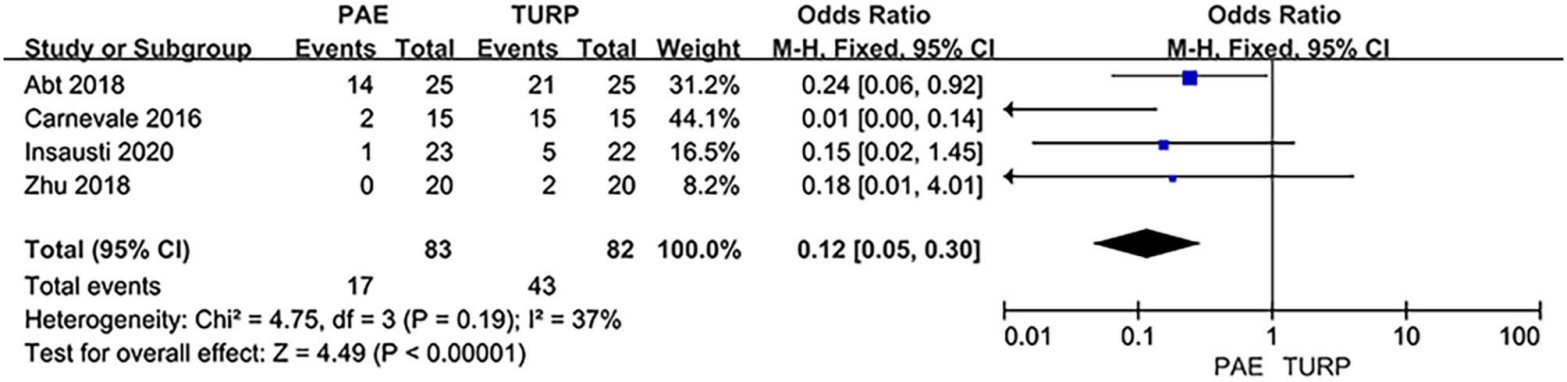
Forest plot for sexual dysfunction.

Six RCTs involving 402 patients were used to analyze the rate of sexual dysfunction. The analysis result revealed no obvious heterogeneity among these studies (*I*^2^ = 37%; *P* = 0.19). The statistical data indicated that the rate of postoperative sexual dysfunction was lower in PAE than TURP (OR 0.12; 95% CI 0.05–0.30; *P* <0.00001; Figure 9).

## Discussion

BPH is one of the most common diseases in men and has a prevalence rate of over 50% of men over 60-years-old, which increases with age (1, 2). Over 1,000 published cases of PAE showed efficacy in curing LUTS caused by BPH, side effects were fewer and recovery times were short (20, 21), since PAE was first reported to treat BPH-LUTS in 2000 (9). We performed a meta-analysis to compare the efficacy and safety of PAE vs. TURP.

The pooled data demonstrated postoperative reduced PV and postoperative increased Qmax were more significant in TURP. It means that TURP had smaller PV and higher Qmax compared with PAE. It may be caused by the different mechanisms of the two procedures. Mechanical obstruction of the urinary tract in prostatic hyperplasia is mainly due to enlargement of the prostatic size, with the protruding prostate tissue then obstructing the urethra. Direct excision of pathologically hyperplastic prostate tissue could instantaneously relieve mechanical obstruction of the urinary tract and achieve satisfactory urodynamics outcomes. However, PAE cannot significantly reduce PV over a short period of time, and it takes a long time to obtain histopathological changes after it disrupts the prostate's blood supply. Malling et al. (22) showed that the peak of PV reduction after PAE was in the 6 month. Carnevale et al. (12) and Zhu et al. (14) showed that the decrease of PV in PAE group needs a longer time. However, Abt et al. (7) followed up for only 3 months, which may exaggerate the advantages of TURP in smaller PV and higher Qmax. Abt et al. (16) revealed that reduction of prostate volume as measured by magnetic resonance imaging was less pronounced after PAE than after TURP after following up for 2 years. Furthermore, the different standards and types of PAE may also influence the efficacy evaluation. Moreover, heterogeneity was rather high for PV and Qmax. Heterogeneity may be related to different types of study designs and surgical approach.

Our study revealed that the rate of postoperative sexual dysfunction was lower in PAE than TURP. Preservation of sexual function is an important point for many BPH patients, and sexual function should be preserved as much as possible during treatment. Gu et al. (23) and Chen et al. (24) showed that postoperative ED was cause by damage of erectile nerve, bleeding, and the heating effect of the electrode during TURP. And Favilla et al. (25) reported that ED may be associated with the cavernous nerve injury caused by electrocoagulation, fibrosis, or thrombosis of the cavernous arteries. However, another common organic factor of ED was penile arterial insufficiency (26). Wang et al. (27) and Bilhim et al. (28) revealed that the incidence of abnormal connections between prostatic arteries and penile arterial in BPH cases, respectively, was 8.8% and 24–43.3%. Therefore, postoperative ED may be associated with untargeted embolization during the PAE procedure. However, Zong et al. (29) described that retrograde ejaculation could be spared if the prostatic tissue just beside and proximal to the verumontanum is preserved. Erectile dysfunction impacts QoL, so a lower sexual dysfunction rate in PAE group may direct the choice of therapy in favor of PAE.

However, our study also demonstrated no available difference in IPSS, QoL, PSA, and PVR, which suggested that the amelioration in IPSS, QoL, PSA, and PRV is similar in TURP group and PAE group. That is different from many other studies. Xu et al. (30) and Zumstein et al. (31) showed that amelioration of IPSS and QoL were better in TURP than in PAE. Gabriel et al. (32) and Xiang et al. (33) reported significant differences between PAE and TURP for Qmax, prostate volume, and PSA. However, Jiang et al. (34) revealed that amelioration of IPSS was similar and amelioration of QoL was better in PAE than in TURP. Neither RCTs had a registered a priori protocol, giving rise to potential bias such as selective outcome reporting and multiple testing. This could be why their studies included some non-RCTs with confounding bias. In the above three studies, two RCTs published by Gao and Carnevale are limited by their defects. We also found that inclusive criteria were different among the enrolled studies after reviewing the criteria. The initial IPSS score of included patients in Carnevale's trial (12) and Gao's trial (13) were ≥ 19 and 7, respectively, and the initial IPSS score in Abt's study (7, 16) and Insausti's study (15) were ≥8. Therefore, patient selection bias due to different inclusive criteria may be an influence on the result. Second, the type of surgery is also an important factor. There are two types of PAE and TURP: unilateral and bilateral embolization for PAE and monopolar and bipolar TURP. For PAE, bilateral embolization has been reported to be more effective than unilateral embolization (35) although over half of patients treated with unilateral embolization can accomplish amelioration in IPSS, QoL, and Qmax, and stay away from prostatic medication. And Carnevale's study (12) showed better IPSS outcomes with the proximal embolization first, then embolize distal technique, with a mean improvement of 21 points. Third, in the studies we included, postoperative outcomes may vary depending on who performed the surgery. Therefore, these experimental results are also biased, and we need more and more standard RCTs to further verify.

Complication rate is similar in TURP group and PAE group. However, complication rate is different among the included trials. Only Carnevale's trial (12) showed that the complication rate was similar in TURP and PAE, other trials (7, 13–16) showed that the complication rate of PAE was lower. However, Gao's trial (13) has been questioned even though it is the largest RCT published so far, because the authors may have underrated the complications of TURP and exaggerated the clinical results with PAE. Our study showed no significant difference in complications, although most RCTs included showed that PAE had a lower complication rate. This could be because there is no clear standard of complication. In Abt's trial (7) and Insausti's trial (15), some mild side effects like mild hematuria, pain, and fever were regarded as complications so the number of complications is more than the number of the subjects and the data cannot be analyzed by Review 5.3. Therefore, well-designed multicenter RCTs with clear criteria will be significant for the efficacy and safety evaluation of PAE and TURP.

Although TURP is still the gold standard, for BPH patients who do not want to have surgery or with operational contraindications, PAE could replace TURP as an alternative treatment. But assessment of radiation exposure with PAE was not included in our analysis. Abt's trial (7) revealed that radiation exposure level of PAE can be below the thresholds recommended by national public health offices. Nevertheless, we must consider radiation exposure of PAE. Andrade et al. (36) showed that radiation exposure of PAE is highly variable. And for anesthesia, prostate embolization is performed under local anesthesia. For frail patients, local anesthesia is a safer form of anesthesia, and it can reduce the risks associated with general anesthesia.

Our meta-analysis included only six RCTs, which reduced selection bias to some extent. However, a few limitations still existed. First, the sample size of the study was too small. Six RCTs included a total of only 402 participants. Among them, only 30, 40, and 45 people were included in Carnevale's trial, Zhu's trial, and Insausti's trial, respectively. Even the largest trial (13) has been questioned by Bilhim (37) because the authors may have underestimated the side effects of TURP and exaggerated the great results with PAE. Second, follow-up time is short. Abt's trial (7) had only 3 months of follow-up data, which made a difference to our results. In our study, maximum follow-up in the included studies is 2 years (13, 16). Pisco et al. (20) revealed that clinical success was 76.3% at long-term (up to 6.5 y) follow-up. Third, the heterogeneity of the assessed outcomes was rather high. The reasons for high heterogeneity are as follows: the disparate embolization standards (like the difference of embolic materials and size of embosphere), the different type of embolization (such as unilateral or bilateral embolization), and the diverse type of TURP (such as monopolar or bipolar technique). This difference may lead to higher heterogeneity and affect the evaluation of therapeutic efficiency. Therefore, we require more elaborate trials with longer follow-up times to assess the efficacy and safety of PAE and TURP. For future randomized controlled trials, first, there should be identical patient inclusion criteria and the initial scores of patients should be basically similar. Second, the type of surgery the patient underwent should be the same as the person who performed it. Third, there should be a clear standard for complications. Fourth, the follow-up time should be long enough.

## Conclusions

In conclusion, although PAE was inferior to TURP in the improvement of PV and Qmax, PAE had lower sexual dysfunction rate. PAE could replace TURP as an alternative treatment for BPH patients who do not want to have surgery or with operational contraindications, although TURP is still the gold standard.

## Data Availability Statement

The original contributions presented in the study are included in the article/supplementary material, further inquiries can be directed to the corresponding author/s.

## Author Contributions

LL and ZG: conception and design. TC: collection and assembly of data. ZX, ZZ, and YM: data analysis and interpretation and manuscript writing. LL: revision of manuscript. All authors contributed to the article and approved the submitted version.

## Funding

This study was supported by the Natural Science Foundation of Shandong Province, Youth Foundation (ZR2020QH186), Yantai Science and Technology Bureau (2020YD002), Shandong Provincial Natural Science Foundation, China (Grant No. ZR2017LH016), and Yantai Science and Technology Bureau (2018SFGY117).

## Conflict of Interest

The authors declare that the research was conducted in the absence of any commercial or financial relationships that could be construed as a potential conflict of interest.

## Publisher's Note

All claims expressed in this article are solely those of the authors and do not necessarily represent those of their affiliated organizations, or those of the publisher, the editors and the reviewers. Any product that may be evaluated in this article, or claim that may be made by its manufacturer, is not guaranteed or endorsed by the publisher.

## Abbreviations

PAE: prostatic artery embolization
TURP: transurethral resection of prostate
RCTs: randomized controlled trials
MD: Median mean
CI: confidence interval
PV: prostate volume
Qmax: maximum flow rate in free uroflowmetry
OR: odds ratio
IPSS: International Prostate Symptoms Score
Qol: quality of life
PVR: post void residual
PSA: prostate-specific antigen
BPH: Benign prostatic hyperplasia
LUTS: lower urinary tract symptoms
5-ARIs: 5-alphareductase inhibitors
EAU: European or American urology
OP: open prostatectomy
NICE: National Institute for Health and Care Excellence.
